# Formulation of Strain Fatigue Criterion Based on Complex Numbers

**DOI:** 10.3390/ma14051227

**Published:** 2021-03-05

**Authors:** Tadeusz Łagoda, Karolina Głowacka, Marta Kurek, Dariusz Skibicki

**Affiliations:** 1 Faculty of Mechanical Engineering, Opole University of Technology, 45-271 Opole, Poland; t.lagoda@po.edu.pl (T.Ł.); k.glowacka@po.edu.pl (K.G.); 2 Faculty of Mechanical Engineering, UTP University of Science and Technology, 85-796 Bydgoszcz, Poland; darski@utp.edu.pl

**Keywords:** normal strain, shear strain, fatigue criteria, critical plane, complex number

## Abstract

In the case of many low-cycle multiaxial fatigue criteria, we encounter a mathematical problem of adding vectors of normal and shear strains. Typically, the problem of defining an equivalent strain is solved by weighting factors. Unfortunately, this ignores the fact that these vectors represent other physical quantities: the normal strain is a longitudinal strain, and the shear strain is a rotation angle. Therefore, the goal of the present work was to propose a method of combining different types of strains by adopting a system of complex numbers. The normal strain was defined as the real part and the shear strain was defined as the imaginary part. Using this approach, simple load states, such as pure bending and pure torsion, have been transformed into an expression for equivalent strain identical to the previously proposed criteria defined by Macha.

## 1. Introduction

Multiaxial fatigue criteria for complex loading conditions have been proposed for decades. Unfortunately, no universal and practically useful solution had been found until now. Typically, stress, strain, and stress–strain, including energy criteria, are used. Stress criteria are the most used models in both monotonic loading and fatigue. These models are also preferred by engineers for the fatigue life estimation of structural components. In the case of loads within low cycle fatigue (LCF), strain criteria are usually the most common. These criteria are the expressions of the equivalent value of the strain. The most-used strain criteria include those formulated in the critical plane, which are based on the normal and shear strain. These quantities are uneven vectors, and so adding them as scalars is an important mathematical problem. Many authors have successfully applied these criteria, but rarely has anyone considered the physical sense of adding such vectors, especially as one type of strain is linear (translation), and the other is the angle of rotation.

Since Cardano provided algebraic solutions to cubic equations in 1545, complex numbers have had many practical applications in physics and engineering. In this paper, complex numbers are used in a similar sense as they are used in cosmology to express distances in four-dimensional spacetime. Time, although it is a dimension expressed in units of time, is reduced to spatial dimensions due to the introduction of imaginary time. Then, despite the qualitative difference between “dimensions”, the distance in four-dimensional spacetime can be easily calculated.

The goal of this work is to propose an expression for the equivalent strain, which is under the mathematical principles of adding vectors representing the same physical quantity. In the present work, we assumed that the strain in the plane was a complex number defined in such a way that the normal strain was the real part of this number, and the shear strain was the imaginary part. This solution has previously been successfully applied to the stress criteria [1]. This work is a continuation of this solution; however, this time, in the strain formulation where additional mathematical inaccuracies appear.

## 2. Strain Multiaxial Fatigue Criteria in Critical Plane

For the low cycle range, multiaxial fatigue criteria based on strain are the most popular as mentioned earlier. Most multiaxial fatigue criteria are recorded in terms of stress. However, this description cannot be used for low cycle fatigue. In such a situation, strain formulation is used, or less frequently energy formulation, i.e., stress–strain. The most commonly used models are those defined in the critical plane, although not always. Below is a selection of them, which are mostly used in the description of multiaxial fatigue using normal and shear strain. There are not as many of these models as the stress ones; however, some of them are used and can be found in the most recent works, in which these models are described in more detail, among others [2,3,4,5,6,7].

The non-linear Kandil–Brown–Miller (*KBM*) relationship [8] can be formulated as

(1) $$KBM=\Delta \gamma_{\eta max}^{j}+S\varepsilon_{\eta }^{j}$$

where as the linear relationship of Lohr–Ellison (*LE*) [9] in the analogous form to (1) is written as

(2) $$LE=\Delta \gamma_{\eta max}^{*}/2+S\varepsilon_{\eta ,a}^{*}$$

where $\Delta \gamma_{\eta max}^{*}$ is the maximum range of the shear strain and the amplitude of normal strain at the shearing plane at an angle of π/4 to the outer surface of the material.

Socie and others [10,11] proposed to additionally consider the mean value but only the stress one:

(3) $$So=\gamma_{eq,a}=\gamma_{\eta s,a}+\varepsilon_{\eta ,a}+\frac{\sigma_{n,m}}{E}$$

Andrews’ criterion [12] is

(4) $$An=\gamma_{eq,a}=\gamma_{amax}+\frac{2S\varepsilon_{n,a}}{1+B}$$

where $\varepsilon_{An}$ is the normal strain amplitude at the plane of the maximum range of the shear strain amplitude and is a criterion analogous to *LE* (2); however, there are two material constants in it.

The Huber–Mises criterion in the strain formulation is generally not placed in the critical plane. However, on this basis, Shang, Wang [13] proposed the criterion of

(5) $$HM=\varepsilon_{eq,a}=\sqrt{\varepsilon_{\eta ,a}^{2}+\frac{1}{3}\frac{\gamma_{max}^{2}}{4}}$$

Here, it can be seen that *KBM* (1) and *HM* (5) are non-linear criteria due to their components. The *LE* (2), *So* (3), and *An* (4) criteria, on the other hand, are linear due to the strain components. All expressions for the equivalent strains (1)–(5) are defined for cyclic loading and recorded in amplitudes, ranges, or maximum values.

## 3. Multiaxial Strain Fatigue Criterion Using to the Concept of Macha

**Figure 1:**
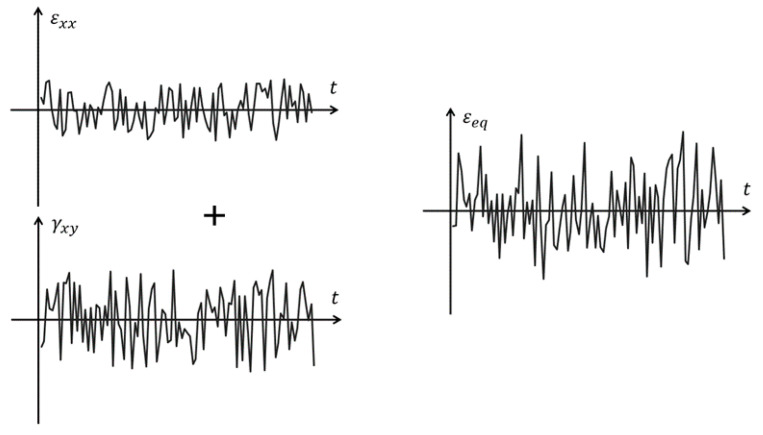
Time history of strain.

Macha [14] defined the model of maximum normal and shear strain in the critical plane to be applied in the case of multiaxial random strain (Figure 1). Therefore, this criterion is more general in nature and can be applied to both cyclical loads as expressed by the Formulas (1)–(5) and random loads.

The criterion has been formulated accepting the following assumptions:

1. Fatigue fracture develops under the influence of a linear combination of the time histories of the normal strain *ε*_ƞ_(*t*) and the shear strain *γ*_ƞ*s*_(*t*)/2 occurring in the S direction in the critical plane with normal ƞ as follows.
  (6) $$b\gamma_{\eta s}\left(t\right)/2+k\varepsilon_{\eta }\left(t\right)$$
2. The S-direction in the fracture plane coincides with the mean direction of the shear strain *γ*_ƞ*s*_(*t*).
3. For a given fatigue life, the maximum value of Equation (6) in the critical plane as shown in Figure 1, under multi-axial random load conditions, fulfils the equation
  (7) $$\underset{t}{\max}\left\{b\gamma_{\eta s}\left(t\right)/2+k\varepsilon_{\eta }\left(t\right)\right\}=q$$
  where *b*, *k*, and *q* are the material constants for a particular form of Equation (7).

This relationship is linear due to the strain components. A paper [15] demonstrated that the criteria dedicated to random loads must be linear due to the strain state components. Therefore, criterion (7), as mentioned above, is a generalization of criteria (2)—*LE*, (3)—Therefore, and (4)—*An*. However, the non-linear criteria (1)—*KBM* and (5)—*HM* cannot be generalized in the same way. The fracture plane orientation is determined by the mean values of principal stress directional cosines. There are three methods for determining the expected critical plain direction: weight functions, variance, and damage accumulation.

In the works [16,17], the expression of Macha (Equation (7)) was detailed and specified in the form of the strain criterion in the plane of normal strain and formulated as

(8) $$OL1=\varepsilon_{eq}\left(t\right)=b\gamma_{\eta s}\left(t\right)/2+\underset{t}{\max}\left\{\varepsilon_{n}\left(t\right)\right\}$$

Selecting the critical plane by the maximum value of the normal strain vector makes this hypothesis useful for brittle materials. Parameter *b* is determined based on the best compliance of the predictions in the case of inconsistent loads in the phase. This parameter can be most accurately determined when performing tests and calculations for the phase shift π/2.

The second proposal for a detailed Macha model determined the critical plane by the shear strain vector of the maximum value and was formulated as

(9) $$OL2=b\underset{t}{\max}\{\gamma_{\eta s}\left(t\right)/2\}+k\varepsilon_{\eta }\left(t\right)$$

In the plane of maximum shearing, the value for an equivalent expression takes the form [16]

(10) $$\varepsilon_{eq}\left(t\right)=\frac{2}{1-\nu }\left(1-\frac{\varepsilon_{af}}{\gamma_{af}}\left(1+\nu \right)\right)\varepsilon_{n}\left(t\right)+2\frac{\varepsilon_{af}}{\gamma_{af}}\varepsilon_{ns}\left(t\right)$$

where the time history of normal and shear strain is defined in multiaxial loading in an elasto-plastic regime, such as

(11) $$\varepsilon_{\eta }(t)={\hat{l}}_{\eta }{}^{2}\varepsilon_{\mathrm{xx}}(t)+{\hat{m}}_{\eta }{}^{2}\varepsilon_{\mathrm{yy}}(t)+{\hat{n}}_{\eta }{}^{2}\varepsilon_{\mathrm{zz}}(t)+2{\hat{l}}_{\eta }{\hat{m}}_{\eta }\varepsilon_{\mathrm{xy}}(t)+2{\hat{m}}_{\eta }{\hat{n}}_{\eta }\varepsilon_{\mathrm{yz}}(t)+2{\hat{n}}_{\eta }{\hat{l}}_{\eta }\varepsilon_{\mathrm{zx}}(t)$$

and

(12) $$\begin{array}{c} \varepsilon_{\eta s}(t)={\hat{l}}_{\eta }{\hat{l}}_{s}\varepsilon_{\mathrm{xx}}(t)+{\hat{m}}_{\eta }{\hat{m}}_{s}\varepsilon_{\mathrm{yy}}(t)+{\hat{n}}_{\eta }{\hat{n}}_{s}\varepsilon_{\mathrm{zz}}(t)+\left({\hat{l}}_{\eta }{\hat{m}}_{s}+{\hat{m}}_{\eta }{\hat{l}}_{s}\right)\varepsilon_{\mathrm{xy}}(t)+\\ +\left({\hat{m}}_{\eta }{\hat{n}}_{s}+{\hat{n}}_{\eta }{\hat{m}}_{s}\right)\varepsilon_{\mathrm{yz}}(t)+\left({\hat{n}}_{\eta }{\hat{l}}_{s}+{\hat{l}}_{\eta }{\hat{n}}_{s}\right)\varepsilon_{\mathrm{zx}}(t). \end{array}$$

For the rectangular coordinate system Oxyz, the mean values of the directional cosines of vectors $\vec{\eta }$ and $\vec{s}$ can be represented as

(13) $${\hat{l}}_{\eta }=\cos(\bar{\eta },x),\;{\hat{m}}_{\eta }=\cos(\bar{\eta },y),\;{\hat{n}}_{\eta }=\cos(\bar{\eta },z),$$

(14) $${\hat{l}}_{s}=\cos(\bar{s},x),\;{\hat{m}}_{s}=\cos(\bar{s},y),\;{\hat{n}}_{s}=\cos(\bar{s},z).$$

If the elasticity is assumed, we obtain

(15) $$\varepsilon_{eq}\left(t\right)=\frac{2}{1-\nu }\left(1-\frac{\sigma_{af}}{2\tau_{af}}\right)\varepsilon_{n}\left(t\right)+2\frac{\sigma_{af}}{\tau_{af}\left(1+\nu \right)}\varepsilon_{ns}\left(t\right)$$

The material parameters *b* and *k* in Formula (9) were defined in Formulas (1) and (11), respectively, as functions of the fatigue limits in the strains and stresses for the elastic body model. Therefore, to use these expressions for equivalent strain, it is necessary to know the fatigue characteristics for the two simple tension–compression (alternating bending) and shear (symmetrical torsion) load states. These formulas can only be directly applied at the fatigue limit level or in the case of parallel characteristics. Otherwise, iterative methods should be used as presented in the papers [18,19].

Macha introduced the concept of strain, which is equal to half of the shear strain, which can be written as

(16) $$\varepsilon_{ns}\left(t\right)=\frac{\gamma_{ns}\left(t\right)}{2}$$

and appears, among others, in Formulas (10) and (15).

**Figure 2:**
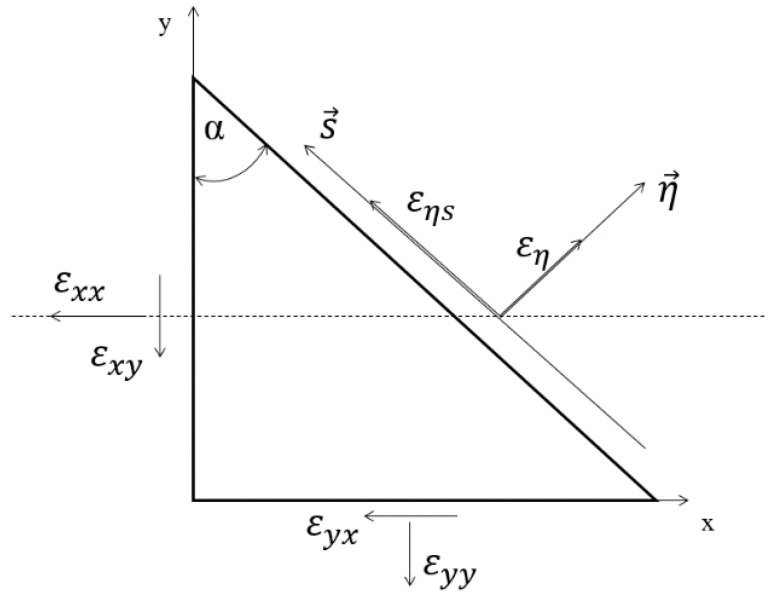
The interpretation of the critical plain orientation [1].

**Figure 3:**
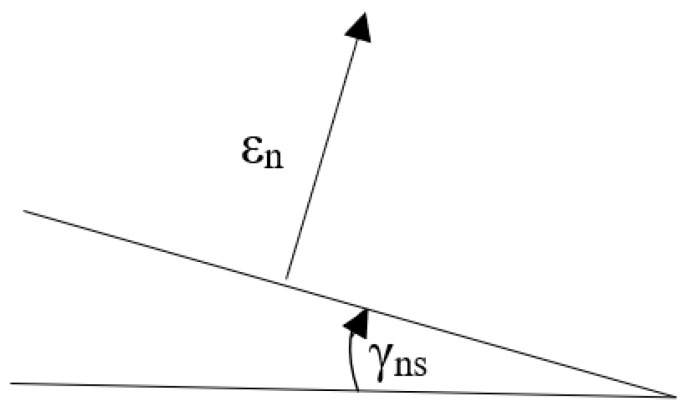
Addition of linear strain and the angle of rotation in the critical plane.

In Figure 2, both active (nominal) strains are shown: normal *ε*_xx_(*t*) and shear *ε*_xy_(*t*). In the critical plane defined by an angle of α, these strains are directed at each other at a right angle. However, this approach has several disadvantages. First, there is a mathematical doubt about adding mutually perpendicular vectors, because these strains are vectors. Thus, from one side, the question arises as to what quantity we obtain as a result. From the other side, this proposal was successfully applied according to this criterion and the other critical plane criteria with both the normal and shear strain taken into account [16,20,21,22,23,24,25,26,27,28]. The approach illustrated in Figure 2 has one more fundamental flaw. Namely, the normal strain (*ε*_xx_, *ε_n_*) is a longitudinal strain, and the shear strain (*γ_xy_*, *γ_n_*) is an angle of rotation. Therefore, the question arises as to what is achieved by adding the linear strain and the angle of rotation as shown in Figure 3 as it is defined in Equations (1) and (15) in the critical plane. This question is still relevant if we consider that this angle is relatively small and fits into small strains.

## 4. Multiaxial Fatigue Criterion in the Critical Plane Using the Complex Numbers Formulation and Discussion of Obtained Proposal

The equation for the equivalent strain according to the proposed multiaxial random fatigue model can be rewritten using complex numbers in the general equation as

(17) $$\varepsilon_{eq}\left(t\right)=\left|p\left(t\right)\right|\left(Acos\varphi +Bisin\varphi \right)$$

where

(18) $$\left|p\left(t\right)\right|=\sqrt{\varepsilon_{x}^{2}\left(t\right)+0.25\gamma_{xy}^{2}\left(t\right)}$$

is the value of the equivalent strain module, *φ* is the phase in a given moment of time, and A and B are material constants.

**Figure 4:**
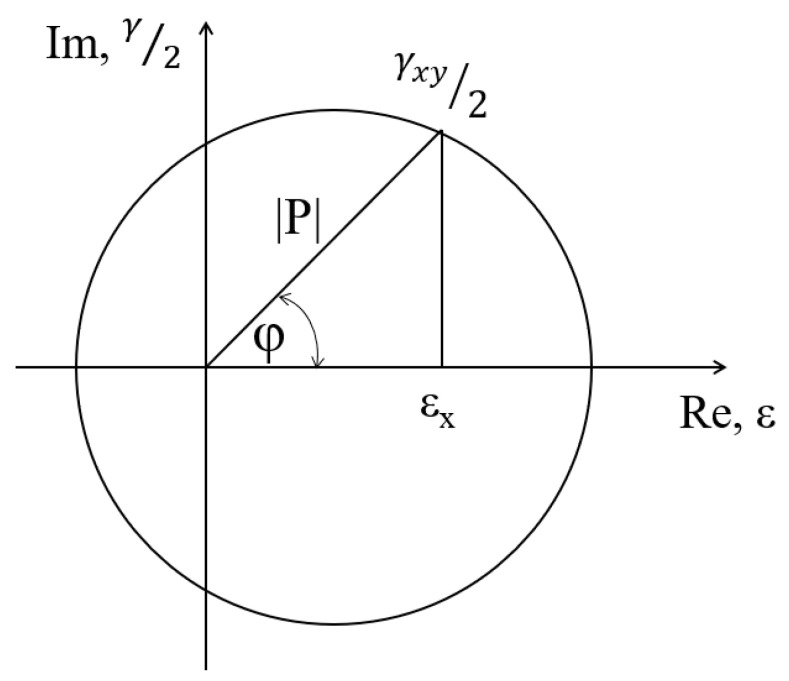
Interpretation of the components of strain in the critical plane as a complex number.

A graphic interpretation of the expression (18) is shown in Figure 4.

In the proposed criterion, the equivalent strain is assumed as a complex number, where the value of the normal strain is the real part and the value of half of the shear strain is the imaginary part. The formula must be corrected for simple states of loading, i.e., for pure tension–compression (alternating bending) and pure shear (symmetrical torsion). Due to this, we obtained a system of equations and two parameters, *A* and *B*, which appear in Equation (17), and can be easily determined.

**Figure 5:**
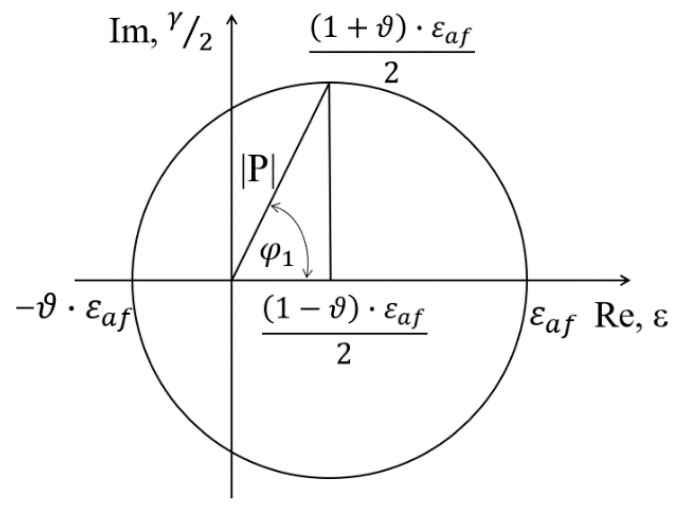
Strain in the critical plane as a complex number for tension [1].

**Figure 6:**
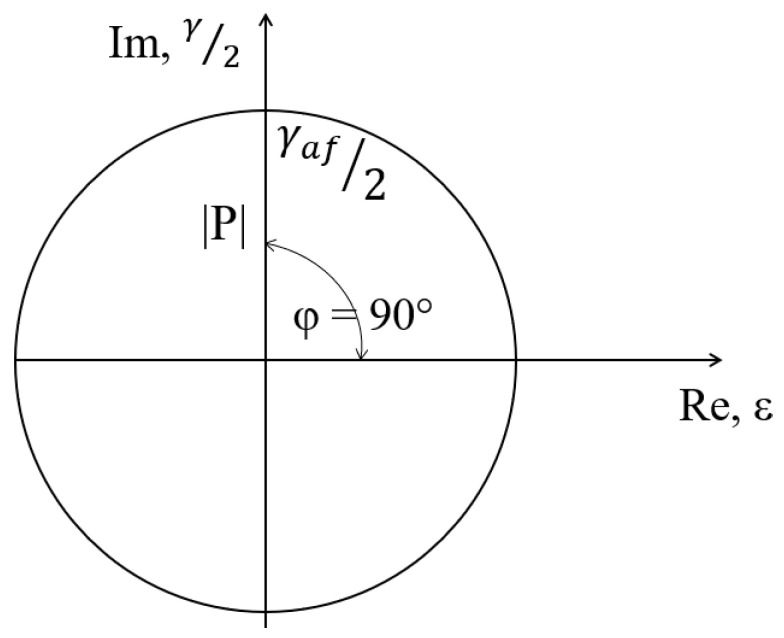
Strain in the critical plane as a complex number for shearing [1].

For the case of tension–compression (or alternating bending) as shown in Figure 5, from Formula (17), we obtain

(19) $$\varepsilon_{eq}\left(t\right)=\left|\varepsilon \left(t\right)\right|\left(A+Bi\right)$$

and, in the case of pure shearing (torsion) based on Figure 6 and Formula (13), we obtain

(20) $$\varepsilon_{eq}\left(t\right)=\left|\gamma \left(t\right)/2\right|\left(Bi\right)$$

Transforming the system of Equations (19) and (20), expressing the quantities represented there as amplitudes, and assuming the fatigue limit for tension–compression as *ε_af_* and shear as *γ_af_*, we obtain an equation equal to Formula (10). Therefore, we concluded that the derived expression for the form of equivalent deformation for multi-axis fatigue loads in terms of complex numbers in the Formula (17) was identical to the Macha proposal (9). Thus, we demonstrated that the mathematical compatibility of the earlier proposal by Macha indicated that the derivation based on complex numbers provided this agreement. Macha’s position was already verified several times in theory and on the basis of experimental research [16,17].

## 5. Conclusions

Based on a short review of the literature, many fatigue criteria formulate an equivalent strain by adding mutually perpendicular normal and shear strain vectors. Such an approach, despite the lack of mathematical justification, produces good results in fatigue life and strength predictions. This is no different when using a criterion based on Macha’s concept; however, the application of the calculus of complex numbers restores its mathematical correctness. The paper demonstrated that treating the strain in the critical plane as a complex number, with the assumption that the normal strain is the real part and half of the shear strain is the imaginary part, led to an expression equal to Macha’s proposal. Owing to this, the model can be successfully developed and applied.

## Data Availability

Data sharing not applicable. No new data were created or analyzed in this study. Data sharing is not applicable to this article.

## Abbreviations

List of Symbols	
Oxyz	rectangular coordinate system
*a*	amplitude
*b*, *k*, *q*, *j*, *A*, and *B*	material constants in multiaxial criteria
*E*	module of elasticity
*eq*	equivalent value
*max*	maximum value
*t*	time
Δ	range
*γ*	shear strain
*ε*	normal strain
*ν*	Poisson value
